# Suitable Areas for Apiculture Expansion Determined by Antioxidant Power, Chemical Profiles, and Pesticide Residues in *Caldcluvia paniculata* Honey and Beeswax Samples

**DOI:** 10.3390/insects13010031

**Published:** 2021-12-28

**Authors:** Enrique Mejías, Carlos Gómez, Tatiana Garrido

**Affiliations:** 1 Centro de Tecnologías Nucleares en Ecosistemas Vulnerables, División de Investigación y Aplicaciones Nucleares—Comisión Chilena de Energía Nuclear, Nueva Bilbao 12501, Santiago 7600713, Chile; 2 Departamento de Química Inorgánica y Analítica, Facultad de Ciencias Químicas y Farmacéuticas, Universidad de Chile, Dr. Carlos Lorca Tobar 964, Santiago 8391063, Chile; cvgomez@gmail.com (C.G.); tgarrido@ciq.uchile.cl (T.G.)

**Keywords:** honey, beeswax, apiculture, pesticides, antioxidants, phenols

## Abstract

**Simple Summary:**

Honey is biologically desirable for antioxidant powers and antiradical capacities. However, pesticide use in farming means that any nearby beehives might become contaminated with undesirable and often harmful compounds. Apart from considerations for bee and human health, producing pesticide-free honey is economically important for Chile, the primary export market of which is the regulation-strict European Union. In the present study, honey and beeswax samples were collected from the Los Lagos Region of Chile and subjected to chemical profiling (phenol contents via Folin–Ciocalteu method; antioxidant power via Ferric Reducing Antioxidant Power Assay (FRAP) antiradical activity via 2,2-Diphenyl-1-picrylhydrazyl Assay (DPPH) and evaluations for pesticide residues (via HPLC-MS/MS and GS-MS).

**Abstract:**

Forty-two samples of Tiaca Honey (*Caldcluvia paniculata*) obtained from beehives belonging to 14 apiaries (three honey samples per apiary) were collected at the end of January near Osorno (40°34′ S, 73°8′ W), Puyehue (40°40′ S, 72°37′ W) and Frutillar 41°7′ S, 72°59′ W) covering an area of 1240 km^2^. They presented the highest phenol contents (0.36 mg gallic acid equivalent/kg) and antioxidant power (1.27 mM equivalent of Fe^+2^/g of sample), and were among the highest for antiradical activity. Phenol contents and antioxidant power (r = 0.72, *p*-value < 0.01) and total phenol contents and antiradical activity (r = 0.69; *p*-value < 0.01) displayed linear correlations. Only two beeswax samples showed residues of the pesticide fenhexamid. The respective sites (Purranque [40°55′ S, 73°10′ W] and Coligual [40°49′ S, 72°54′ W]) were the only areas located near active farms. Additionally, the *m*/*z* value 163.1091 was found as an element to identify honeys. Data were used to construct a mapped suitability index ranking for pesticide-free areas with high biological quality. The provided chemical profiles will aid local beekeepers in obtaining international certifications, particularly for the EU market. In turn, the constructed maps indicate suitable areas for apiculture expansion, while differentiated pesticide detection in honey and beeswax requires further comparative research.

## 1. Introduction

Pesticides play an important role in farming, with primary positive benefits, such as pest control, helping to improve crop yields [1,2]. However, pesticides also present several widely described negative effects for the environment and human health [3,4,5,6]. Estimates indicate that more than 98% of insecticides do not reach their destination [7], which is why these products are frequently found in the water, soil, atmosphere, and farmed crops [8].

Insecticide use has shifted over the last 20 years from organophosphates and carbamates to neonicotinoids [9]. Neonicotinoids are acetylcholine agonists that bind to nicotinic acetylcholine receptors, thereby triggering continuous signaling and causing insect death [10]. Due to the high persistence of pesticides in the environment, these compounds can be transferred to honey and other apicultural products either directly or indirectly by bees during production [11,12,13,14]. A lack of regulations and appropriate oversight have resulted in an indiscriminate use of pesticides, thus potentiating the lethal effects for populations that should not be exterminated, such as bees [15]. For this pollinating insect, the median lethal dose (LD50) of neonicotinoids is 0.003–0.006 μg/bee via oral ingestion [16]. Furthermore, damage is induced in proportion to the amount of insecticide [17]. Sublethal neonicotinoid doses consequently provoke nervous system disorders in bees that result in disorientation, memory loss, behavioral changes, communication difficulties, and an inability to carry out pollinating functions [9,18,19]. Neonicotinoids can also cause immunodeficiency, which is one of the causes for colony collapse disorder [20]. In the same way, pesticides residues detected in bee product samples offer a wide spectrum of risk for health of consumers, from slight allergenic reaction after exposure to carcinogenic effect [21]. Although, there are several sources of pollution, in many instances the presence of varroacides compounds is related to beekeeping regular activities, and thus, it is up to appropriate application of products. This explains the differences among honeys produced in the same apiary and samples from one country to another [22,23].

The European Union (EU) is one of the primary worldwide importers of honey and is the main market for Chilean honey, with 96% of national honey exports destined for EU countries [24]. The EU is one of the most stringent markets in terms of sustainable production and is particularly concerned about the negative consequences of pesticides, among other farming practices [25]. In 2013, the European Food Safety Authority identified at least three high-risk neonicotinoids for bees, especially regarding colony survival and development: clothianidin, imidacloprid, and thiamethoxam. As such, use of these insecticides is restricted by the EU [26,27,28].

It is worth highlighting that in addition to neonicotinoids, other pesticides used in farming, such as organophosphates, can also affect bees and, consequently, honey production [29]. In the interest of sustainable apicultural practices, current research interests in the area include determining the possible routes through pesticides affect honey, beeswax, bee pollen, and propolis, as well as establishing if there is a relationship of any effects with farming activities near hives [30]. Therefore, the objectives of this study were to (i) characterize the phenolic, antioxidant, antiradical profiles, calories and content of total carbohydrates, and ashes of honey samples. Likewise, pesticides residues in honey and beeswax samples and (ii) use the obtained data to establish suitable geographical areas in Chile for the pesticide-free production of honey and other biologically valuable apicultural products.

## 2. Materials and Methods

### 2.1. Honey and Beeswax Samples

The sampled beehives (n = 14) were in the Los Lagos Region of southern Chile (39°16′ S to 44°04′ S). Three samples of honey and a single sample of beeswax were collected from each beehive. All samples were collected during the summer (January–February 2016). Immediately after collection, the samples were transported to the laboratory for posterior analyses. Information regarding bee deaths in the colonies was gathered through interviews with local beekeepers.

### 2.2. Mellisopalynological Analysis for Determining the Botanical Origin of Honey Samples

The botanical composition of honey samples was quantitatively counted following methods described by Louveaux, Maurizio, and Vorwohl (1978) [31]. Briefly, honey (20 g) was placed on acetolyzed slides (Montenegro, Gómez, Díaz-Forestier, and Pizarro, 2008) [32]. Then, a sample aliquot was diluted with warm distilled water (20 mL at 40 °C), and the solution was transferred to an appropriate tube and centrifuged at 3500 rpm for 10 min. The supernatant was discarded, and the pollen residue was deposited at the bottom of the tube for resuspension in distilled water (100 µL). An aliquot (20 µL) was then taken and added to a slide together with Calberla’s solution (10 µL), which was either basic fuchsine or diamond. The slide was gently dried. Finally, melted glycerinated gelatin (15 µL) was added to the mixture. For each sample, pollen grain residues were identified using an optical microscope at 400 and 1000× magnifications.

### 2.3. Preparation of Honey Solutions

First, honey samples (50 g) were mixed with distilled water (100 mL) acidified with HCl (pH = 2). The mixture was placed in a volumetric flask, and water was added until reaching a final volume of 250 mL. The extract was then filtered with cotton. Phenolic compounds were separated by column chromatography using the Amberlite XAD-2 resin (250 mm height, 20 mm diameter, 2 mL/min drop speed; Sigma-Aldrich, St. Louis, MO, USA). The column was washed with acid water (100 mL, pH = 2) and, subsequently, neutral distilled water (200 mL). Phenolic compounds were eluted with methanol p.a EMSURE^®^ Merck Darmstadt, Germany (300 mL) The phenolic extract was collected and concentrated in vacuo to dryness at 45 °C. The dry residue was resuspended in distilled water (5 mL). The suspension was put in a decantation funnel, and diethyl ether (5 mL) was added. The organic phase was collected and washed twice with diethyl ether p.a EMSURE ^®^ Merck (5 mL). The solution was concentrated to dryness in vacuo at 45 °C. The residual was resuspended in 2 mL of high-performance liquid chromatography (HPLC) grade methanol (Merck LiChrosolv Darmstadt, Germany), filtered (0.45 μm pore size), and stored at −20 °C until analysis. The extract was weighed prior to storage.

### 2.4. Colorimetric Assays for Determining Total Phenolic Compounds

The procedure described by Singleton and Rossi [33] and Buratti et al. [34] was used with minor modifications. Briefly, honey solution (200 µL) was mixed with the Folin–Ciocalteu reagent (50 µL—Merck KGaA, Darmstadt, Germany) and, subsequently, 20% Na_2_CO_3_ (150 µL). Distilled water was then added to a total volume of 1 mL. Absorbance was read at 765 nm after 30 min in a DLab SP—UV 1000 spectrophotometer (Beijing, China). Gallic acid (Sigma-Aldrich, St. Louis, MO, USA) was used as a standard to derive the calibration curve (0–150 mg/mL). The results defined the phenolic contents, which were expressed as the g equivalent of gallic acid/kg of sample.

### 2.5. Ferric Reducing/Antioxidant Power (FRAP) Assays for Determining Antioxidant Power

FRAP assays were performed according to Bertoncelj et al. [35]. The FRAP reagent was prepared by mixing 2,4,6-Tris(2-pyridyl)-s-triazine Sigma-Aldrich (2.5 mL; 10 mM 2,4,6-Tris(2-pyridyl)-s-triazine/40 mM of HCl) with 20 mM FeCl_3_ (2.5 mL). Finally, 0.3 M acetate buffer (25 mL, pH = 3.6) was added to the mixture. The FRAP reagent was prepared just prior to each assay run. Antioxidant power was determined by mixing honey solution (0.2 mL) with the FRAP reagent (1.8 mL). Absorbance was read at 593 nm after 10 min. FeSO_4_ 7H_2_O was used as a standard to derive the calibration curve (50–1000 mM). Values were expressed as the mM equivalent of Fe^+2^/g of sample. Assays were performed at room temperature.

### 2.6. Determinations of Antiradical Activity

The procedure described by Meda et al. [36] and modified by Mejías and Montenegro [37] was followed to determine antiradical activity. The 1,1-diphenyl-2-picrylhydrazyl radical DPPH (Calbiochem, Darmstadt, Germany) assay was used to determine the antiradical properties of the chemical compounds in honey by inhibiting or decreasing the oxidant activity of DPPH. For this, honey solutions (750 µL) were mixed with the DPPH radical (1.5 mL) in methanol (0.02 mg DPPH/mL MeOH). Absorbance was read at 517 nm after 15 min. A blank sample was prepared with methanol. Ascorbic acid (Calbiochem, Darmstadt, Germany) was used as a standard to derive the calibration curve (1–10 mg/mL). The values for antiradical activity were expressed as mg equivalent of ascorbic acid/g of sample.

### 2.7. Determination of Total Carbohydrate Content in Honey Samples

The total carbohydrate percentage of each honey was previously measured by refractometry (*w*/*w* percentage). Next, 15 g of honey were weighed and mixed with 10 mL of water. The pH of the resulting solution was adjusted to 1.0 by adding HCl 1.2 M from an automatic titrator, provided by a combined pH electrode. The total carbohydrate percentage was then reduced to a final value of 40.0%, *w*/*w*, by dilution with acidified water at pH 1.5. The total carbohydrate percentage of pure honeys is in average 80%, *w*/*w* [38].

### 2.8. Determinations of Total Ash Content in Honey Samples

The ash content was determined according to the methods of AOAC 2000 [39] with modifications. First, 10 g of honey were placed in combustion pots, which required preheating to darkness with a gas flame to prevent honey foaming. Thereafter, the samples were incinerated at 600 °C in a burning muffle for 5 h. After cooling at room temperature, the obtained ash was weighed.

### 2.9. Direct Sample Analysis-Time of Flight-Mass Spectrometry (DSA-TOF-MS) for Determining Chromatography Profiles

For direct sample analysis-time of flight-mass spectrometry, HPLC grade water containing 0.1 M NaOH was added to honey (500 mg). The liquid sample (10 µL) was placed in a mesh holder for analysis. Assays were run on a direct sample analysis-time of flight-mass spectrometry (DSA-TOF-MS, Perkin Elmer, Waltham, MA, USA) using the following conditions: corona current = 30 µA; heater temperature = 300 °C; auxiliary gas (N_2_) flow = 4 L/min; nebulizer gas (N_2_) pressure = 80 psi; drying gas (N_2_) flow = 3 L/min; and drying gas (N_2_) temperature = 25 °C. The DSA-TOF-MS was run in positive ionization mode with a flight tube voltage of −10,000 V. The capillary exit voltage was set to 100 V for normal MS analysis and 155 V for collision induced dissociation analysis. Mass spectra were acquired with a mass range of 100–3000 *m*/*z* and acquisition rate of 1 spectra/s. To maintain mass accuracy, five lock mass ions were used (*m*/*z* 121.0509, *m*/*z* 622.0299, *m*/*z* 922.0119, *m*/*z* 1521.9771, and *m*/*z* 2121.9405). All samples were analyzed for only 10 s.

### 2.10. Extraction Methodologies

#### 2.10.1. Quick, Easy, Cheap, Effective, Rugged, and Safe (QuEChERS)

QuEChERS extraction was performed following methodology proposed by Barganska et al. [11], with certain modifications. Briefly, honey (2 g) samples were dissolved with 15 mL of a solution of 1% acetic acid in acetonitrile. This mixture was transferred to the Extraction Tube containing the salt kit provided with the Dispersive QuEChERS (DisQuE) was added (Cat. No. 176001903; Waters, Milford, MA, USA). The composition of this kit included 4 g MgSO4, 1 g NaCl, 1 g trisodium citrate dehydrate, and 0.5 g disodium hydrogen citrate sesquihydrate. Internal standards (50 μL; triphenyl phosphate 100 μg/mL) were subsequently added to the mixture. The samples were shaken vigorously for 1 min and centrifuged at 4400 rpm for 5 min. Samples were further cleaned by transferring the obtained supernatant (4 mL) to a dispersive sample preparation extraction tube, which was then shaken for 45 s. Thereafter, the tube was centrifuged at 5000 rpm for 2 min. The resulting supernatant was used for chromatographic analysis. Samples were cleaned using MgSO_4_ (150 mg), primary–secondary amine (PSA; 25 mg), and a C18 (PSA) sorbent (25 mg). The above methodology was also applied to beeswax samples, excepting the dissolution of beeswax (1 g) with chloroform (5 mL) in the initial steps.

#### 2.10.2. Solid-Phase Extraction

Pesticides were extracted and identified from honey samples using the methodology proposed by Bohm et al. [40], with modifications. Briefly, honey samples (2 g) were homogenized with a citrate buffer solution (10 mL, pH 4.0). After agitation for 15 min, the mixture was centrifuged (4000 × RPM, 5 min, 5 °C) and filtered. The entire supernatant was transferred to the Oasis HLB 3 cc Vac Cartridge (Cat. No. WAT 094226; Waters, Milford, MA, USA) for sample preparation extraction on a vacuum station previously conditioned with MeOH (6 mL) and water (6 mL). The extracts were rinsed and then eluted with a solution (5 mL) containing 3% formic acid in MeOH. Finally, the elutions were concentrated to dryness. Dry residues were reconstituted in a mobile phase solution A (750 µL; see below for details) and filtered for further analysis via liquid chromatography tandem-mass spectrometry (LC-MS/MS). Beeswax samples (2 g) were dissolved by vigorous agitation with 3:1 chloroform/MeOH (5 mL). The homogenized solution was mixed with 96% MeOH (5 mL) and centrifuged. The entire supernatant was transferred onto an OASIS HLB cartridge. All subsequent steps for beeswax processing were the same as with honey processing.

### 2.11. Chromatography

A total of 242 pesticides from the following groups were analyzed: organochlorines, organophosphates, carbamates, thiocarbamates, pyrethroids, and neonicotinoids. Regarding neonicotinoids, acetamiprid, imidacloprid, thiamethoxam, and thiacloprid were considered (Supporting Material Table S1).

#### 2.11.1. LC-MS/MS Analysis

Honey and beeswax were analyzed by UPLC–MS/MS using a XEVO Triple Quadrupole Tandem Mass Spectrometer (ACQUITY UPLC H-Class System; Waters Corp., Milford, MA, USA). Separation was facilitated by using an Acquity- Ethylene Bridged Hybrid (BEH) C18 column (1.7 lm, 2.1 9 50 mm; Waters Corp., Milford, MA, USA). A mobile phase gradient was composed of solutions A and B. Solution A was comprised by 5 mM of 10% ammonium formate in 10% methanol and 90% HPLC grade water. Solution B was comprised by 5 mM ammonium formate in 90% methanol and 10% HPLC grade water. The oven temperature was 30 °C, with an injection volume of 10 µL. The following MS/MS parameters were used: ionization mode = positive; scan type = MRM; dwell-time = 20 ms; ion spray voltage = 5.500 V; source = 300 °C; and analysis time = 21 min.

#### 2.11.2. Gas Chromatography–Mass Spectrometry (GC-MS) Analysis

Chromatography analyses were conducted in an Agilent 7890A GC-MS (Santa Clara, CA, USA) with solvent vent-mode injection using a programmable temperature vaporization inlet with the 5975C Mass Selective Detector (Agilent, Santa Clara, CA, USA). Chromatography conditions were as follows: injector temperature = 250 °C; column temperature = 40 °C for 5 min, then increased to 240 °C at a speed of 3 °C/min, and finally 240 °C for 10 min; carrier gas = helium at 20 mL/min flow rate; and column = Zebron ZB-5ms 30 m × 0.24 mm × 0.25 mm (Phenomenex, Torrance, CA, USA). Mass detector conditions were as follows: transference line temperature = 260 °C; ionization trap temperature = 17 °C; ion impact energy = 70 eV; and analysis time = 37.5 min. The used pesticide standards were from Dr. Ehrenstorfer GmBH (Augsburg, Germany). Stock solutions were prepared at a concentration of 400 µg mL^−1^ in ethyl acetate and were stored at −20 °C in amber vials. All solvents and reagents used were HPLC grade (Merck Millipore, Darmstadt, Germany).

### 2.12. Suitability Index

The values obtained for each analyzed honey sample in relation to total phenols, antioxidant power, and antiradical activity were separately calculated in decreasing order for each georeferenced location. From this, an increasing scale of 1 to 3 was used to rank each variable from lowest to highest. The remaining numbers in the series were calculated proportional to the following parameterization (e.g., for total phenols [TP]):

$${\mathrm{TP}}_{\mathrm{new}\;\mathrm{scale}}=\frac{{\mathrm{TP}}_{\mathrm{original}\;\mathrm{scale}}-\min\left({\mathrm{TP}}_{\mathrm{original}\;\mathrm{scale}}\right)}{\max\left({\mathrm{TP}}_{\mathrm{original}\;\mathrm{scale}}\right)-\min\left({\mathrm{TP}}_{\mathrm{original}\;\mathrm{scale}}\right)}\times \left(3-1\right)+1$$

where ${\mathrm{TP}}_{\mathrm{new}\;\mathrm{scale}}$ is expressed in the original units of measurement for total phenols, i.e., g equivalent of gallic acid/kg and min (${\mathrm{TP}}_{\mathrm{original}\;\mathrm{scale}}$) was defined as the smallest Total Phenolic value observed among all honeys.

This new parameterization methodology was applied to obtain values for total phenols (TP), antioxidant power (AP), antiradical activity (AA), Total Carbohydrate Content (TC) and Total Ash (TA). Additionally, there is a set of Energy values obtained from TC. TC may be alternatively replaced by Energy when these data are available. To construct a suitability index for each location, the respective scores were added in such a way that sample XX met the following:

Sample XX: [(X(TP); Z(AP); Y(AA); W(TC);T(TA)] where 1 < T, W, X, Y, Z < 3

Finally, the suitability index (SI) for location *i* was calculated as follows:

$${\mathrm{SI}}_{i}=\sum^{\;}(T_{i}+W_{i}+X_{i}+Y_{i}+Z_{i})\;\;\;\;\;\;\;\;\;5\leq {\mathrm{SI}}_{i}\leq 15$$

### 2.13. Statistical Analysis

All assays for each honey and beeswax sample were performed in triplicate. An exploratory analysis of the data was conducted to evaluate assumptions of normality and to select appropriate statistical methodologies. All calculations and map constructions were performed in the R v.3.2.5. software (2016) with the ggmap and ggplot2 packages. Furthermore, all statistical analyses followed methodological guidelines for reproducible research using the knitr library. The source code in R can be requested from the corresponding author.

For the comparison between the different honeys, an analysis of variance (ANOVA) was carried out for each of the variables studied. Tukey’s multiple comparisons test was carried out to evaluate the statistical significance of the differences between honeys. The assumptions of the ANOVA test were corroborated by residual analysis: normality, independence and homoscedasticity. The statistical significance of the correlations was evaluated using Pearson’s correlation test. The normality of the data was assessed using Shapiro’s test and QQ normality plots.

## 3. Results

### 3.1. Botanical Origin and Chemical Analyses of Honey Samples

The botanical origins of the studied honey samples are indicated in Table 1, which also shows the percentage of the three most predominant botanical species found in analyses.

**Figure 1:**
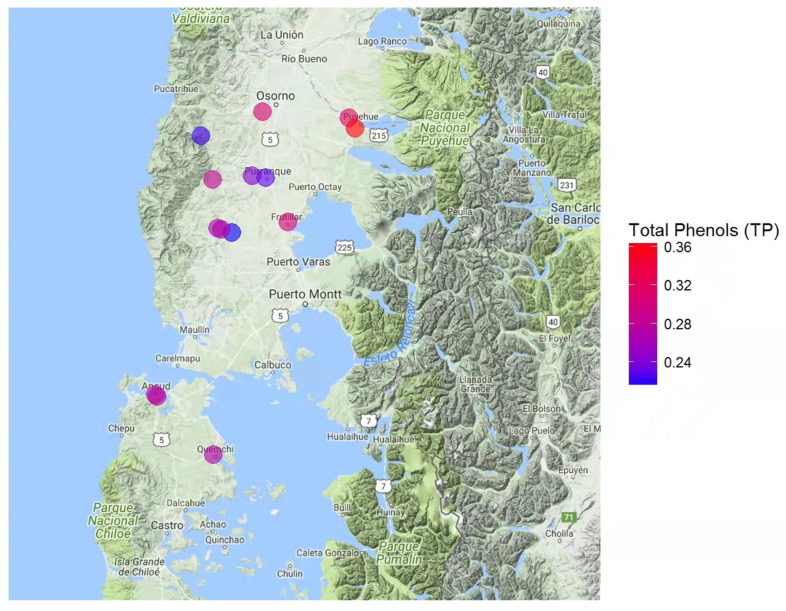
Map of the Los Lagos Region (Chile, 39°16′ S to 44°04′ S) indicating the sampled hive locations and respective phenol contents in honey samples. Contents concentration is indicated by a color scale going from blue (lower contents) to red (higher contents).

**Figure 2:**
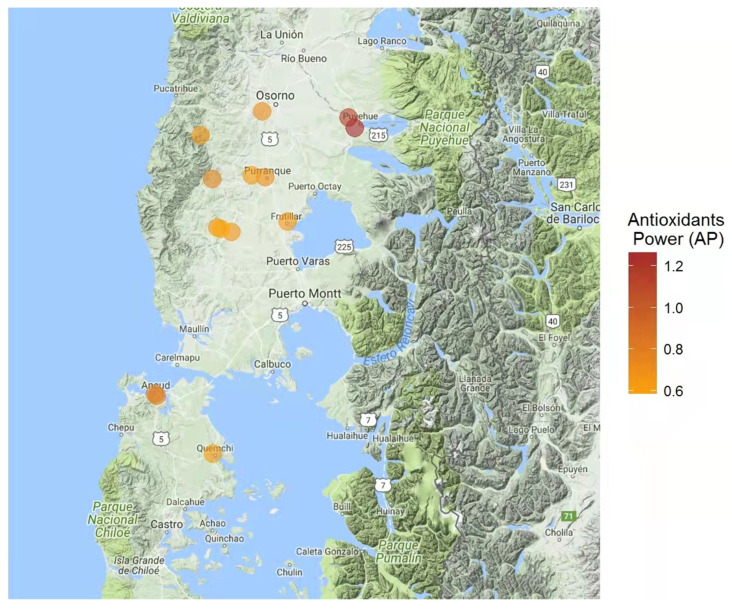
Map of the Los Lagos Region (Chile, 39°16′ S to 44°04′ S) indicating the sampled hive locations and respective antioxidant power of honey samples (mg of ascorbic acid equivalents/g of sample). Antioxidant power is indicated by a color scale going from light orange (lower power) to dark orange (higher power).

**Figure 3:**
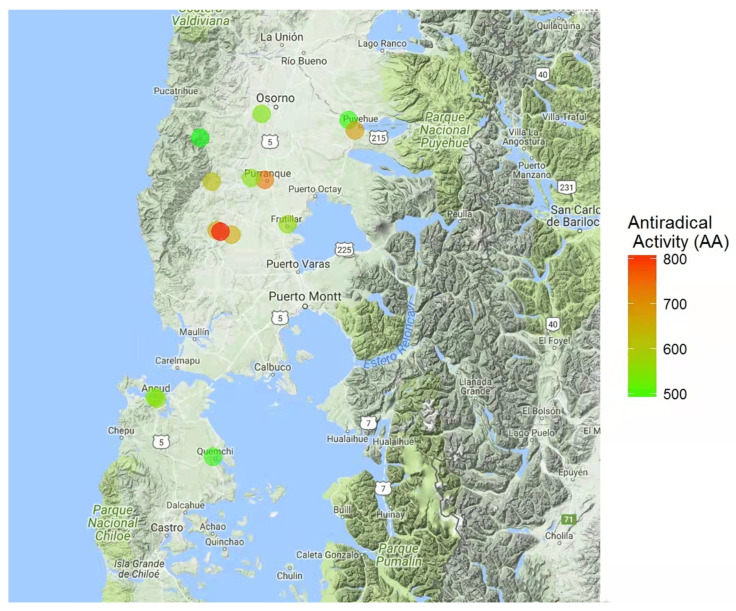
Map of the Los Lagos Region (Chile, 39°16′ S to 44°04′ S) indicating the sampled hive locations and respective antiradical activity of honey samples. Antiradical activity is indicated by a color scale going from green (lower activity) to red (higher activity).

Overall results for total phenol contents, antioxidant power, and antiradical activity are shown in Table 2. The index of each component was also calculated for posterior suitability index determinations for the assessed areas. More specifically, total phenol contents were established based on a gallic acid standard (i.e., mg gallic acid equivalent/kg of sample; Figure 1). Honey sample E, collected in proximity to Puyehue (40°40′ S, 72°37′ W), presented the highest phenol contents (i.e., 0.36 mg/kg). Similarly, honey sample E presented the highest antioxidant power (1.27 mm equivalent of Fe^+2^/g of sample; Figure 2), as established by FRAP analyses. Finally, antiradical activity was measured as the ability to inhibit or decrease the oxidizing effect of DPPH (Figure 3). Honey samples E, F, and L, respectively located in proximity to Puyehue (40°40′ S, 72°37′ W), Purranque (40°55′ S, 73°10′ W), and Fresia (41°09′ S, 73°27′ W), had the highest antiradical activities.

Notably, results for phenol contents, antioxidant power, and antiradical activity coincided for honey sample E (Table 3, Figure 1, Figure 2 and Figure 3). Furthermore, positive linear correlations were found among all evaluated honeys for total phenol contents and antioxidant power (r = 0.72, *p*-value < 0.01), as well as for total phenol contents and antiradical activity (r = 69; *p*-value < 0.01). These results suggest that biological antioxidant activity primarily depends on the phenol contents of the honey sample, which would be inherited from the predominant nectar-containing plants, as established through botanical origin analyses (Table 1).

### 3.2. MS Analysis of Honey Samples

Direct sample analysis-time of flight-mass spectrometry for the collected honey samples was performed between 100 and 3000 *m*/*z*. Signals were principally distributed between 100 and 350 *m*/*z*, and the calibration process resulted in residues with less than 0.0006 *m*/*z*. The calibrating volume used during analysis was 20 µL. Direct sample analysis is a source of ambient ionization. Ambient mass spectrometry can sample and ionize analyte molecules directly from surfaces with little to no preparation. Direct sample analysis operates on the principles of atmospheric pressure chemical ionization. Therefore, *m*/*z* values obtained via direct sample analysis-time of flight-mass spectrometry can be interpreted as honey fingerprints showing a distribution pattern that should be related to botanical origin.

In this study, six signals were regularly detected for the honeys analyzed. However, three of these were observed without exception in all honeys. Likewise, the obtained mass spectrometry signals for the control sample differed from honey samples A–N. For example, an *m*/*z* value of 105.0708 was detected in honey samples A–N, but not in honey sample Z (control). The same case was noted for *m*/*z* values of 322.0545. In addition to this, the signal corresponding to the *m*/*z* value 163.1093 was observed both in the selected honeys and in the control sample. This suggests the presence of a signal that could be an element to identify honeys independently of their geographical origin (Table 2).

**Figure 4:**
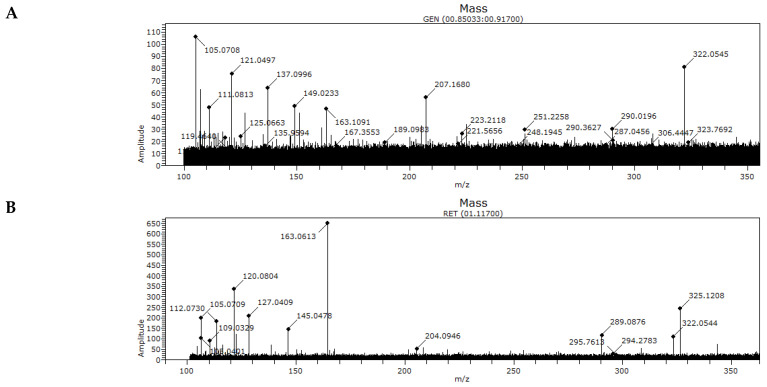

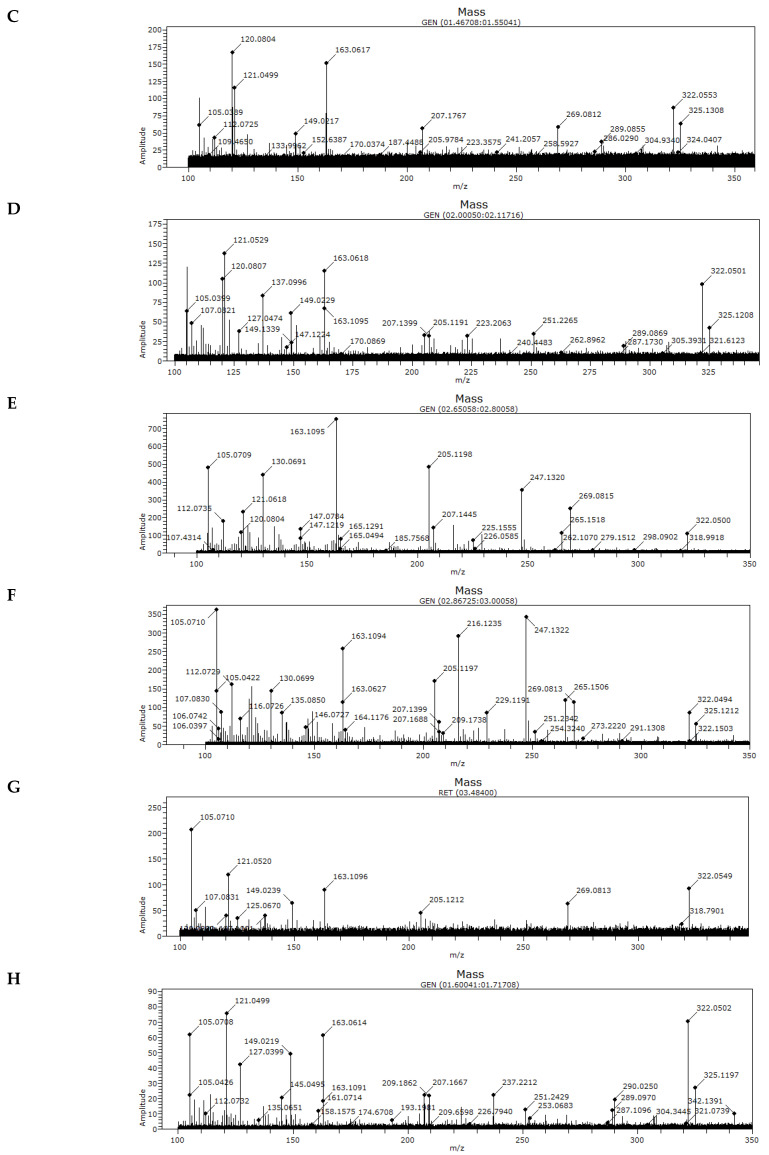

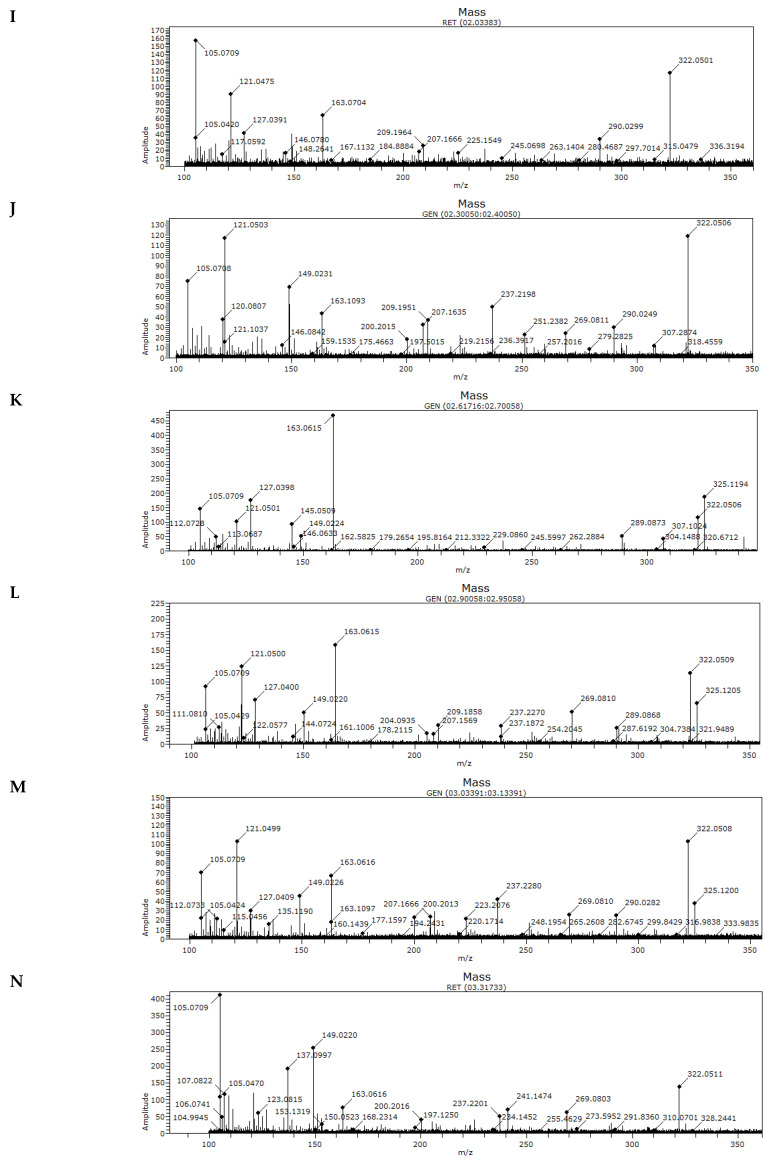

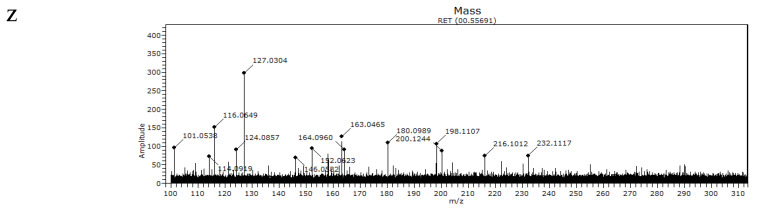
Spectroscopic profiles for phenolic compounds in 14 honey samples (**A**–**N**) obtained from the Las Lagos Region (Chile, 40°15′ S to 44°14′ S), as well as in 1 sample (**Z**) obtained from the Araucanía Region (Chile, 37°35′ S to 39°37′ S).

For example, honey samples A-N originated from the same region and harvested in the same period, and all contained the evergreen species *Caldcluvia paniculata* (Figure 4). The *m*/*z* values also indicated distribution similarities among certain samples, such as honey samples C-D-E-J, which were collected from proximal beehives (Figure 4). These samples showed a presence of *C. paniculata* (Table 1) and similar composition percentages for the other two identified botanical species (i.e., *Luma/Myrceugenia and Weinmannia trichosperma*).

In turn, honey sample Z was collected as a control from the Araucanía Region (38°45′ S 72°40′ W), located approximately 310 km from the other sampled beehives. The obtained mass spectrometry signals for the control sample differed from honey samples A-N.

### 3.3. Presence of Pesticides in Honey/Beeswax Samples

**Figure 5:**
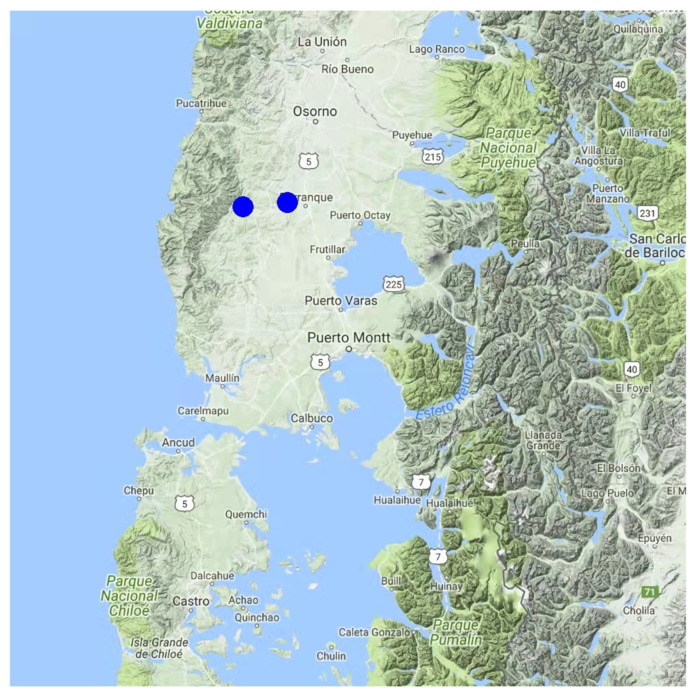
Map of the Los Lagos Region (Chile, 39°16′ S to 44°04′ S) indicating the two hive locations where beeswax samples tested positive for the pesticide fenhexamid.

No residues of the 242 evaluated pesticides were found for 14 of the 16 assessed beehives. However, fenhexamid was detected in beeswax samples collected in proximity to Purranque (40°55′ S, 73°10′ W) and Coligual (40°49′ S, 72°54′ W) (Figure 5). These two localities were the only sites with nearby farming activities (e.g., berries and raps).

### 3.4. Suitable Areas for the Pesticide-Free Production of Honey and Beeswax

**Figure 6:**
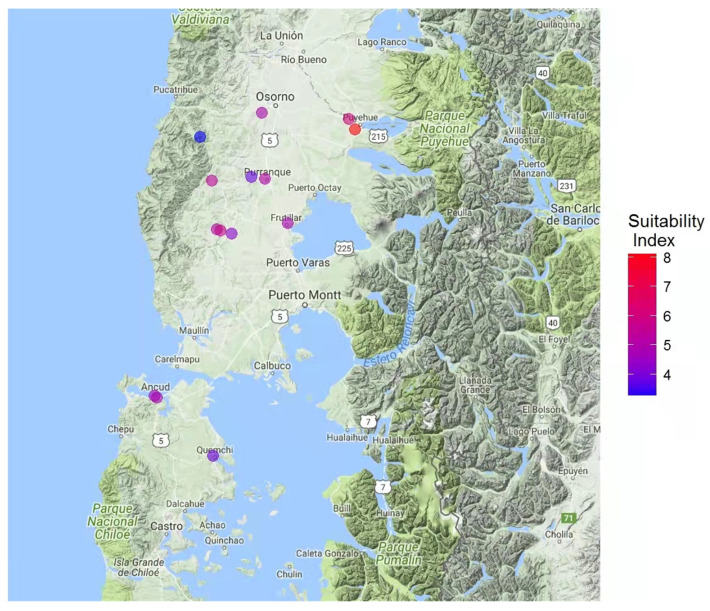
Map of the Los Lagos Region (Chile, 39°16′ S to 44°04′ S) indicating the most suitable areas for apiculture. The suitability index is indicated by a color scale going from blue (more suitable) to red (less suitable).

From the chemical profile and pesticide residue results, as well as information provided by beekeepers, a map was constructed indicating the most suitable zones for apicultural activities in the Los Lagos Region of Chile (Figure 6). Area suitability for honey and beeswax production was established based on biological attributes, indicators of quality, and the absence of pesticides (Table 3). The area proximal to Puyehue (40°40′ S, 72°37′ W) presented a suitability index value of 7.95, which was significantly higher than the other assessed areas. Honey and beeswax samples from this area also had the highest total phenol contents and antiradical activities (Figure 1 and Figure 2), as well as a lack of pesticide residues (Figure 5).

## 4. Discussion

The highly variable environments of the Los Lagos Region (Chile) give rise to wide floral diversity. Distinguishable among this diversity are two large plant formations, temperate laurel forests (Wintero-Nothofagetea) and sub-Antarctic deciduous forests (Nothofagetea pumilionis antarcticae) [41]. Laurel forests are composed of three forest subtypes: Valdivian or evergreen forests (38–43° S), north-Patagonian forests (43–47° S), and sub-Antarctic forests (47–55° S) [42]. Environmental floral components include species of liverworts, mosses, ferns, and gymnosperms, such as *Pilgerodendron uviferum* (Guaitecas cypres), *Podocarpus nubigenus* (Chilean podocarp), *Saxegothaea conspicua* (female maniu), *Drimys winteri* (winter’s bark), *Eucryphia cordifolia* (Ulmo), *Gevuina avellana* (Chilean hazel), *Laureliopsis philippiana* (tepa), *Luma apiculata* (Chilean myrtle), *Nothofagus* sp. (coihue), *Berberis buxifolia* (Magellan barberry), *Pernettya* sp. (chaura), and *Ugni molinae* (Chilean guava), among others; as well as monocotyledon species such as *Philesia magellanica* (Chilean bellflower), *Chusquea quila* (colihue cane), *Luzuriaga* sp. (coralito), *Carex* sp. (sedges), *Codonorchis lessonii* (field lily), *Juncus* sp. (rushes), and *Uncinia* sp. (clin-clín), among others [43]. Nearly all of these species are endemic to the Los Lagos Region, meaning unique representation in south-central Chilean Patagonia and the extreme south of Argentina [42]. Other species found in the humid woodlands of the Chilean and Argentinean Mountain ranges are the native evergreen *C. paniculata* (tiaca) and endemic Cunoniaceae evergreen *W. trichosperma* (tineo), both of which serve as an important source of nectar for honey production [44]. All the assessed samples showed a presence of *C. paniculata*, which is consistent with the geographical origin of the collected honey samples. Similarly, this result coincided with the harvest date (January), which aligned with the peak flowering period for this species in the region where the sampled colonies were located [45].

The chemical composition of honey varies according to the floral origin from which bees collect nectar. Consequently, the properties of honey, such as antibacterial, antioxidant, or antidiabetic activities depend on geographical location and respective flora [37,45,46]. Different chemical compounds have been found in honey and can be related to floral origin, such as volatile aromatic compounds, derived from carotenes; amino acids and respective derivatives; aromatic acids and respective esters; aromatic aldehydes; and phenolic compounds [47]. In fact, many of these phenolic and aromatic compounds are used as markers of floral origin for honey and other apicultural products [44,48,49,50]. This would explain the similarity in spectroscopic profiles obtained for the 14 honey samples collected from the Los Lagos Region and the calibrating variations detected for the control honey sample obtained 310 km from the other colonies (Figure 4).

The antioxidant and antiradical abilities of honey are highly related to the presence and types of phenolic compounds [35,51,52,53]. Furthermore, antioxidant power depends on the number and position of OH- groups present on flavonoid structures [54]. In turn, antiradical ability, evaluated through a FRAP assay, is based on the capacity to reduce Fe^3+^ to Fe^2+^ in the presence of 2,4,6-Tris(2-pyridyl)-s-triazine [55]. Regarding the presently obtained results for these traits, honey samples collected in proximity to Puyehue showed significantly greater total phenol contents (Figure 1) and higher antioxidant power (Figure 2). Furthermore, linear and positive correlations were found between total phenols and antioxidant power (r = 0.72; *p*-value < 0.01) and between total phenols and antiradical activity (r = 0.69; *p*-value < 0.01). These results are indicative of honey quality recommended for human consumption, particularly as oxidative stress would induce cell-level damage, such as lipoperoxidation, protein damage, and nucleic acid, all of which would give rise to biological complications such as carcinogenesis, mutagenesis, aging, and arteriosclerosis [56].

Honey and beeswax samples were tested for 242 pesticides, including organochlorines, organophosphates, carbamates, thiocarbamates, pyrethroids, and neonicotinoids. No traces of these pesticide groups were found in 14 of the evaluated honey samples. However, fenhexamid was detected in two beeswax samples originating from near Purranque and Coligual (Figure 6). Fenhexamid is a widely used fungicide with site-specific actions that inhibit the 3-ketoreductase enzyme, which is involved in C-4 demethylation during the biosynthesis of ergosterol, a cellular membrane component of fungi [57]. This fungicide has an LD50 > 215 µg/bee depending on exposure contact [58]. Fenhexamid is frequently used to control *Botrytis cinerea*, translating into a commonplace presence of this fungicide in farming sectors [57,59,60,61]. This finding aligns with some reports provided by beekeepers with colonies located near sites with a known presence of fenhexamid. Namely, berry farms affected by *B. cinerea* existed in the same area as the bee colonies with detected pesticide residues. Interestingly, although some local beekeepers have reported decreased colony populations, the present results may indicate that this phenomenon might be due to disease in the bees (e.g., varroasis or nosema) rather than an improper use of pesticides; however, bee population can decrease and still not find pesticide residues in honey.

Chilean honey exports have increased in recent years. The primary buyer of this national product is the EU, accounting for 96% of honey exports. This market is very strict regarding sustainable and environmentally friendly productive practices [25]. In the EU, pesticide risks for bees are evaluated according to European and Mediterranean Plant Protection Organization guidelines. Consequently, the use of various pesticides is restricted because of risks to the environment and human health [58]. These economic and regulatory factors highlight the need to identify adequate areas for apiculture in Chile. In that way, the absence of pesticides in all honey samples despite the nearby crops gives good confirmation about good agricultural practices fulfillment [62]. Although in the south of Chile, a great number of native melliferous species are found [63], beekeeping is involved in pollination of fruits, as it occurs in other places along the country and the continent [64]. For this reason, the risk of exposure to one toxic compound or a mixture of them increase concerns of beekeepers [15]. Additionally, changes observed on the original and natural properties of honeys are detected when pesticides are present at the same time in the final content of these samples [65].

## 5. Conclusions

To this end, the present study is the first to provide a suitability index map for apiculture sites in the Los Lagos Region (Figure 6). This map establishes areas free of 242 pesticides and with honey of interesting biological quality. The developed maps and calculated data will aid local beekeepers in obtaining certifications as to the quality and safety of their products. Finally, the differentiated concentration of pesticides in honey and beeswax highlights the need for further comparative studies in order to apply this model to other regions of the country.

## Figures and Tables

**Table 1:** Predominant botanical species (%) found in each honey sample.

Apiary (*)-Total Pollen Grains	*Caldcluvia paniculata*	*Luma*/*Myrceugenia*	*Weinmannia trichosperma*	Other Species
A (1.851)	35 ± 0.02 h	0	0	65
B (1.908)	72 ± 0.02 c	5 ± 0.01 b	6 ± 0.01 b	17
C (2.232)	52 ± 0.03 f	2 ± 0.01 c	0	46
D (2.241)	33 ± 0.02 h	0	0	67
E (1.836)	85 ± 0.04 a	6 ± 0.02 b	6 ± 0.01 b	3
F (1.986)	81 ± 0.04 a	7 ± 0.01 a	5 ± 0.01 b	7
G (1.968)	28 ± 0.03 i	0	0	72
H (1.953)	58 ± 0.02 e	2 ± 0.02 c	2 ± 0.01 c	38
I (2.049)	75 ± 0.01 b	5 ± 0.02 b	5 ± 0.02 b	15
J (2.169)	63 ± 0.02 d	2 ± 0.01 c	2 ± 0.01 c	33
K (1.965)	70 ± 0.03 c	6 ± 0.01 b	7 ± 0.02 a	17
L (2.073)	71 ± 0.02 c	5 ± 0.02 b	6 ± 0.01 b	18
M (2.001)	48 ± 0.03 f	0	0	52
N (1.827)	43 ± 0.03 g	0	0	57

Values represent the mean of triplicate samples. The means reported in the same column are significantly different according to Tukey’s test. (*p* < 0.05) if denoted by these letters. (*). Three honey samples were taken from each apiary. The number of pollen grains corresponds to the sum of the total number of grains of the 3 honeys from each apiary.

**Table 2:** DSA-TOF-MS signals for analyzed honeys. ND: Not Detected.

Sample	*m*/*z*					
A	105.0708	ND	121.0497	163.1091	207.1680	322.0545
B	105.0709	120.0804	ND	163.0613	ND	322.0544
C	105.0389	120.0804	121.0499	163.0617	207.1767	322.0553
D	105.0399	120.0807	121.0529	163.0618	207.1399	322.0501
E	105.0709	120.0804	121.0618	163.1095	207.1445	322.0500
F	105.0422	ND	ND	163.1094	207.1399	322.0494
G	105.0710	ND	121.0520	163.1096	ND	322.0549
H	105.0708	ND	121.0499	163.0614	207.1667	322.0502
I	105.0709	ND	121.0475	163.0704	207.1666	322.0501
J	105.0708	120.0807	121.1037	163.1093	207.1635	322.0506
K	105.0709	ND	121.0501	163.0615	ND	322.0506
L	105.0709	ND	121.0500	163.0615	207.1569	322.0509
M	105.0709	ND	121.0499	163.0616	207.1666	322.0508
N	105.0709	ND	ND	163.0616	ND	322.0511
Z (CONTROL)	ND	ND	ND	163.0465	ND	ND

**Table 3:** Phenol, antioxidant power, and antiradical activity results obtained for honey samples from the Los Lagos Region (Chile).

Sample	Phenols ^†^ ± SD	Antioxidant Power ^‡^ ± SD	Antiradical Activity ^≡^ ± SD	Total Carbohydrates ^Ʇ^ ± SD	Energy ^#^	Total Ash *	Phenol Index	Antioxidant Index	Antiradical Index	Carbohydrate Index	Energy Index	Ash Index	Suitability Index
A	0.222 ± 0.011 a	0.65 ± 0.09 a	485.5 ± 0.01 a	82.1 ± 0.5 a	329 ± 4 a	0.08 ± 0.008 i	1.1	1.3	1.0	2.1	2.1	1.0	6.4
B	0.320 ± 0.008 b	1.15 ± 0.06 b	490.1 ± 0.01 a	83.4 ± 0.9 a	332 ± 4 a	0.19 ± 0.006 b	2.4	2.7	1.0	2.9	2.5	2.8	11.8
C	0.303 ± 0.010 c	0.64 ± 0.06 a	558.5 ± 0.06 b	80.4 ± 0.5 a	321 ± 6 a	0.21 ± 0.008 a	2.2	1.2	1.4	1.0	1.0	3.0	8.8
D	0.272 ± 0.013 d	0.61 ± 0.07 a	492.4 ± 0.02 a	81.5 ± 0.6 a	326 ± 5 a	0.12 ± 0.007 g	1.7	1.2	1.0	1.7	1.7	1.7	7.3
E	0.366 ± 0.011 e	1.27 ± 0.04 b	681.8 ± 0.06 c	83.6 ± 0.6 a	334 ± 4 a	0.15 ± 0.006 d	3.0	3.0	2.2	3.0	2.7	2.2	13.3
F	0.227 ± 0.008 f	0.71 ± 0.07 d	737.5 ± 0.07 d	80.7 ± 0.1 a	323 ± 6 a	0.22 ± 0.008 a	1.1	1.4	2.5	1.2	1.3	3.0	9.3
G	0.239 ± 0.005 g	0.62 ± 0.05 e	546.0 ± 0.03 b	82.5 ± 0.5 a	331 ± 7 a	0.09 ± 0.006 j	1.3	1.2	1.4	2.3	2.3	1.2	7.3
H	0.282 ± 0.010 d	0.79 ± 0.01 d	539.2 ± 0.06 b	83.6 ± 0.8 a	330 ± 6 a	0.17 ± 0.005 c	1.9	1.6	1.3	3.0	2.2	2.5	10.3
I	0.263 ± 0.010 d	0.67 ± 0.08 f	678.5 ± 0.01 c	82.2 ± 0.3 a	327 ± 5 a	0.12 ± 0.007 f	1.6	1.3	2.2	2.1	1.8	1.7	8.9
J	0.308 ± 0.004 h	0.70 ± 0.07 f	536.8 ± 0.01 a	81.8 ± 0.4 a	327 ± 4 a	0.14 ± 0.006 e	2.2	1.4	1.3	1.9	1.8	2.0	8.8
K	0.284 ± 0.005 d	0.77 ± 0.08 g	624.1 ± 0.04 c	81.6 ± 0.7 a	326 ± 7 a	0.16 ± 0.005 d	1.9	1.6	1.8	1.8	1.7	2.3	9.4
L	0.273 ± 0.009 i	0.95 ± 0.06 h	817.5 ± 0.03 e	81.2 ± 0.9 a	323 ± 5 a	0.09 ± 0.007 i	1.8	2.0	3.0	1.5	1.3	1.2	8.4
M	0.265 ± 0.008 d	0.79 ± 0.06 g	560.1 ± 0.06 b	83.5 ± 0.7 a	336 ± 8 a	0.18 ± 0.006 b	1.6	1.7	1.4	2.9	3.0	2.7	10.3
N	0.217 ± 0.004 j	0.66 ± 0.09 c	654.7 ± 0.05 c	81.6 ± 0.5 a	326 ± 5 a	0.12 ± 0.007 h	1.0	1.3	2.0	1.8	1.7	1.7	7.7

^†^ Phenols = equivalent g of gallic acid/kg of sample. ^‡^ Antioxidant Power = equivalent mM of Fe^+2^/g of sample. ^≡^ Antiradical Activity = equivalent mg of ascorbic acid/g of sample. ^Ʇ^ Total Carbohydrates = g/100 g of sample. ^#^ Energy = kcal/100 g of sample. * Total Ash = g/100 g of sample. SD = Standard Deviation. Values represent the mean of triplicate samples. a, b, c, d, e, f, g, h, i, j. The means reported in the same column are significantly different according to Tukey’s test (*p* < 0.05) if denoted by these letters.

## Supplementary Materials

The following are available online at https://www.mdpi.com/article/10.3390/insects13010031/s1, supporting Table S1: List of pesticides analyzed in honey and beeswax samples.
