# Supplementary material for: Suitable Areas for Apiculture Expansion Determined by Antioxidant Power, Chemical Profiles, and Pesticide Residues in Caldcluvia paniculata Honey and Beeswax Samples
Source: Insects. 2021 Dec 28;13(1):31. doi: 10.3390/insects13010031 (PMC8777965; doi:10.3390/insects13010031)
Supplement: Supplementary file 1 [file insects-13-00031-s001.zip › insects-1457343-supplementary.pdf]

Supporting Table S1. List of pesticides analyzed in honey and beeswax samples							
N°	Pesticide	DL	QL	Extraction method		Chromatography method	
				SPE	QuEChERS	LC-MS/MS	GC-MS
1	2,4 D	0,05	0,1	√		√	
2	ABAMECTIN	0,005	0,01	√		√	
3	ACEPHATE	0,005	0,01	√		√	
4	ACEQUINOCYL	0,005	0,01	√		√	
5	ACETAMIPRID	0,005	0,01	√		√	
6	ACETOCHLOR	0,005	0,01		√		√
7	ACRINATHRIN	0,005	0,01		√		√
8	ALACHLOR	0,005	0,01		√		√
9	ALDICARB	0,005	0,01	√		√	
10	ALDICARB SULFONE	0,005	0,01	√		√	
11	ALDICARB SULFOXIDO	0,005	0,01	√		√	
12	ALDRIN	0,005	0,01		√		√
13	ATRAZINE	0,005	0,01		√		√
14	AZADIRACHTIN	0,005	0,01	√		√	
15	AZINPHOS ETHYL	0,005	0,01		√		√
16	AZINPHOS METHYL	0,005	0,01		√		√
17	AZOXYSTROBIN	0,05	0,1	√		√	
18	BAC C10 BENZALKONIUM CHLORIDE	0,005	0,01	√		√	
19	BAC C12 BENZALKONIUM CHLORIDE	0,005	0,01	√		√	
20	BAC C14 BENZALKONIUM CHLORIDE	0,005	0,01	√		√	
21	BENALAXYL	0,005	0,01	√		√	
22	BENOMYL/CARBENDAZIM	0,005	0,01	√		√	
23	BENTAZON	0,005	0,01	√		√	
24	BHC ALPHA	0,005	0,01		√		√
25	BHC BETA	0,005	0,01		√		√
26	BHC DELTA	0,005	0,01		√		√
27	BIFENAZATE	0,005	0,01		√		√
28	BIFENTHRIN	0,005	0,01		√		√
29	BITERTANOL	0,005	0,01		√		√
30	BOSCALID	0,005	0,01	√		√	
31	BRODIFACOUM	0,005	0,01	√		√	
32	BROMACIL	0,005	0,01	√		√	
33	BROMADIOLONE	0,005	0,01		√		√
34	BROMOPHOS ETHYL	0,005	0,01		√		√
35	BROMOPHOS METHYL	0,005	0,01		√		√
36	BROMOPROPYLATE	0,005	0,01		√		√
37	BUPROFEZIN	0,005	0,01		√		√
38	CADUSAFOS	0,005	0,01		√		√
39	CAPTAFOL	0,005	0,01		√		√
40	CAPTAN	0,005	0,01		√		√
41	CARBARYL	0,005	0,01	√		√	
42	CARBOFURAN	0,005	0,01	√		√	
43	CARBOPHENOTHION	0,005	0,01		√		√
44	CARTAP HCL	0,005	0,01	√		√	
45	CHLOFENTEZINE	0,005	0,01	√		√	
46	CHLORANTRANILIPROLE	0,005	0,01	√		√	
47	CHLORDANE CIS	0,005	0,01		√		√

48	CHLORDANE TRANS	0,005	0,01		√		√
49	CHLORDENE	0,005	0,01		√		√
50	CHLORFENAPYR	0,005	0,01		√		√
51	CHLORFENSON	0,005	0,01		√		√
52	CHLORFENVINPHOS	0,005	0,01		√		√
53	CHLOROBENZILATE	0,005	0,01		√		√
54	CHLOROTHALONIL	0,005	0,01		√		√
55	CHLORPYRIFOS ETHYL	0,005	0,01		√		√
56	CHLORPYRIFOS METHYL	0,005	0,01		√		√
57	CYHEXATIN/AZOCICLOTIN	0,005	0,01	√		√	
58	CLETODIM (EYZ)	0,005	0,01		√		√
59	CLOTHIANIDIN	0,005	0,01	√		√	
60	CYANAZINE	0,005	0,01		√		√
61	CYFLUTHRIN (**)	0,005	0,01		√		√
62	CYFLUTHRIN BETA	0,005	0,01		√		√
63	CYHALOTHRIN GAMMA	0,005	0,01		√		√
64	CYHALOTHRIN L	0,005	0,01		√		√
65	CYPERMETHRIN	0,005	0,01		√		√
66	CYPROCONAZOLE	0,005	0,01	√		√	
67	CYPRODINIL	0,005	0,01	√		√	
68	CYROMAZINE	0,005	0,01	√		√	
69	DDAC DIDECYLDIMETHYLAMMONIUM CHLORIDE	0,005	0,01	√		√	
70	DDD op	0,005	0,01		√		√
71	DDD pp	0,005	0,01		√		√
72	DDE op	0,005	0,01		√		√
73	DDE pp	0,005	0,01		√		√
74	DDT op	0,005	0,01		√		√
75	DDT pp	0,005	0,01		√		√
76	DELTAMETHRIN	0,005	0,01		√		√
77	DEMETON-S	0,005	0,01		√		√
78	DIAZINON	0,005	0,01		√		√
79	DICHLOBENIL	0,005	0,01		√		√
80	DICHLOFLUANID	0,005	0,01		√		√
81	DICHLORVOS	0,005	0,01		√		√
82	DICLORAN	0,005	0,01		√		√
83	DICOFOL op (**)	0,005	0,01		√		√
84	DICROTOPHOS (**)	0,005	0,01		√		√
85	DIELDRIN	0,005	0,01		√		√
86	DIFENOCONAZOLE	0,005	0,01	√		√	
87	DIFLUBENZURON	0,005	0,01	√		√	
88	DIMETHENAMID	0,005	0,01		√		√
89	DIMETHOATE	0,005	0,01	√		√	
90	DIMETHOMORF	0,005	0,01	√		√	
91	DIPHENYLAMINE	0,005	0,01		√		√
92	DISULFOTON	0,005	0,01		√		√
93	DODINE	0,005	0,01	√		√	
94	EMAMECTIN BENZOATE	0,005	0,01	√		√	
95	ENDOSULFAN I	0,005	0,01		√		√
96	ENDOSULFAN II	0,005	0,01		√		√
97	ENDOSULFAN SULFATE	0,005	0,01		√		√
98	ENDRIN	0,005	0,01		√		√
99	EPTC	0,005	0,01		√		√

100	ESFENVALERATE/FENVALERATE	0,005	0,01	√		√	
101	ETHION	0,005	0,01		√		√
102	ETHOPROFOS	0,005	0,01	√		√	
103	ETOFENPROX	0,005	0,01	√		√	
104	FENAMIPHOS	0,005	0,01		√		√
105	FENARIMOL	0,005	0,01		√		√
106	FENAZAQUIN	0,005	0,01	√		√	
107	FENBUCONAZOLE	0,005	0,01		√		√
108	FENCLORPHOS	0,005	0,01		√		√
109	FENHEXAMID	0,005	0,01		√		√
110	FENITROTHION	0,005	0,01		√		√
111	FENOXYCARB	0,005	0,01	√		√	
112	FENPROPATHRIN	0,005	0,01		√		√
113	FENPROPIMORF	0,005	0,01	√		√	
114	FENPYROXIMATE	0,005	0,01	√		√	
115	FENTHION	0,005	0,01		√		√
116	FERBAM	0,005	0,01		√		√
117	FIPRONIL	0,005	0,01		√		√
118	FLOCOUMAFEN	0,005	0,01	√		√	
119	FLUAZINAM	0,005	0,01	√		√	
120	FLUDIOXINIL	0,005	0,01	√		√	
121	FLUFENOXURON (**)	0,005	0,01	√		√	
122	FLUMETRALIN	0,005	0,01		√		√
123	FLUQUINCONAZOLE	0,005	0,01		√		√
124	FLUSILAZOLE	0,005	0,01		√		√
125	FLUTRIAFOL (**)	0,005	0,01		√		√
126	FLUTOLANIL	0,005	0,01	√		√	
127	FLUVALINATE (**)	0,005	0,01		√		√
128	FOLPET	0,005	0,01		√		√
129	FONOFOS	0,005	0,01		√		√
130	FORCHLORFENURON	0,005	0,01	√		√	
131	FORMETANATE	0,005	0,01	√		√	
132	FORMOTHION (**)	0,005	0,01		√		√
133	GLUFOSINATE AMONNIUM	0,005	0,01		√		√
134	HALOXIFOP METHYL	0,005	0,01	√		√	
135	HEPTACHLOR	0,005	0,01		√		√
136	HEPTACHLOR EPOXIDE	0,005	0,01		√		√
137	HEPTENOPHOS	0,005	0,01		√		√
138	HEXACHLOROBENZENE	0,005	0,01		√		√
139	HEXACONAZOLE	0,005	0,01		√		√
140	HEXAZINONE (**)	0,005	0,01		√		√
141	HEXYTIAZOX	0,005	0,01	√		√	
142	IMAZALIL	0,005	0,01	√		√	
143	IMIDACLOPRID	0,005	0,01	√		√	
144	INDOXACARB	0,005	0,01	√		√	
145	IPRODIONE	0,005	0,01		√		√
146	ISOFENPHOS	0,005	0,01		√		√
147	KRESOXIM METHYL	0,005	0,01		√		√
148	LENACIL	0,005	0,01		√		√
149	LINDANE	0,005	0,01		√		√
150	LINURON	0,005	0,01	√		√	
151	LUFENURON	0,005	0,01	√		√	
152	MALATHION	0,005	0,01		√		√

153	MANDIPROPAMID	0,005	0,01	√		√	
154	METALAXYL	0,005	0,01		√		√
155	METAMITRON	0,005	0,01	√		√	
156	METAFLUMIZOLE	0,005	0,01	√		√	
157	METHAMIDOPHOS	0,005	0,01	√		√	
158	METHIDATHION	0,005	0,01		√		√
159	METHIOCARB	0,005	0,01	√		√	
160	METHOXYCHLOR	0,005	0,01		√		√
161	METHOXYFENOZIDE	0,005	0,01		√		√
162	METOLACHLOR	0,005	0,01	√		√	
163	METOMYL	0,005	0,01	√		√	
164	METRAFENONA	0,005	0,01	√		√	
165	METRIBUZIN	0,005	0,01	√		√	
166	MEVINPHOS	0,005	0,01		√		√
167	MIREX	0,005	0,01		√		√
168	MONOCROTOPHOS	0,005	0,01	√		√	
169	MYCLOBUTANIL	0,005	0,01		√		√
170	NAPROPAMIDE	0,005	0,01		√		√
171	NOVALURON	0,005	0,01	√		√	
172	NUARIMOL	0,005	0,01		√		√
173	OMETHOATE	0,005	0,01	√		√	
174	OXADIAZON	0,005	0,01		√		√
175	OXAMYL	0,005	0,01	√		√	
176	OXYFLUORFEN	0,005	0,01		√		√
177	PACLOBUTRAZOL	0,005	0,01		√		√
178	PARATHION ETHYL	0,005	0,01		√		√
179	PARATHION METHYL	0,005	0,01		√		√
180	PENCONAZOLE	0,005	0,01	√		√	√
181	PENDIMETHALIN	0,005	0,01		√		√
182	PERMETHRIN	0,005	0,01		√		√
183	PHORATE	0,005	0,01		√		√
184	PHOSALONE	0,005	0,01		√		√
185	PHOSMET	0,005	0,01	√		√	√
186	PHOSPHAMIDON	0,005	0,01		√		√
187	PIRAZOPHOS	0,005	0,01		√		√
188	PIRIMETHANIL	0,005	0,01	√		√	√
189	PIRIMICARB	0,005	0,01		√		√
190	PIRIMIPHOS ETHYL	0,005	0,01		√		√
191	PIRIMIPHOS METHYL	0,005	0,01		√		√
192	PROCHLORAZ	0,005	0,01	√		√	√
193	PROCYMIDONE	0,005	0,01		√		√
194	PROFENOFOS	0,005	0,01		√		√
195	PROPAMOCARB	0,005	0,01	√		√	√
196	PROPARGITE	0,005	0,01		√		√
197	PROPICONAZOLE	0,005	0,01		√		√
198	PROPOXUR	0,005	0,01	√		√	
199	PROPYZAMIDE	0,005	0,01	√		√	
200	PROTIOCONAZOLE	0,005	0,01	√		√	
201	PYMETROZIN	0,005	0,01	√		√	
202	PYRACLOSTROBIN	0,005	0,01	√		√	
203	PYRIDABEN	0,005	0,01		√		√
204	PYRIPROXYFEN	0,005	0,01		√		√
205	QUINALPHOS	0,005	0,01		√		√
206	QUINOMETHIONATE	0,005	0,01		√		√

207	QUINOXIFENO	0,005	0,01		√		√
208	QUINTOZENE	0,005	0,01		√		√
209	ROTENONE	0,005	0,01		√		√
210	SIMAZINE	0,005	0,01		√		√
211	SPINETORAM	0,005	0,01	√		√	
212	SPINOSAD	0,005	0,01	√		√	
213	SPIRODICLOFEN	0,005	0,01	√		√	
214	SPIROTETRAMAT	0,005	0,01	√		√	
215	SULFUR (S8)	0,005	0,01		√		√
216	TEBUCONAZOLE	0,005	0,01	√	√		√
217	TEBUFENOZIDE	0,005	0,01	√		√	
218	TEFLUTHRIN	0,005	0,01	√		√	
219	TERBACIL	0,005	0,01		√		√
220	TETRACONAZOLE	0,005	0,01		√		√
221	TETRADIFON	0,005	0,01		√		√
222	THIABENDAZOLE	0,005	0,01	√		√	
223	THIACLOPRID	0,005	0,01	√		√	
224	THIAMETHOXAM	0,005	0,01	√		√	
225	THIDIAZURON (**)	0,005	0,01	√		√	
226	THIOCYCLAM HYDROGEN OXALATE	0,005	0,01	√		√	
227	THIOPHANATE METHYL	0,005	0,01	√		√	
228	TOLCLOFOS METHYL	0,005	0,01	√		√	
229	TOLYLFLUANID	0,005	0,01		√		√
230	TOXAPHENE (**)	0,005	0,01		√		√
231	TRIADIMEFON	0,005	0,01	√		√	
232	TRIADIMENOL	0,005	0,01	√		√	
233	TRIAZOPHOS	0,005	0,01	√		√	
234	TRICHLORFON (**)	0,005	0,01		√		√
235	TRIFLOXYSTROBIN	0,005	0,01		√		√
236	TRIFLUMIZOLE	0,005	0,01	√		√	
237	TRIFLUMORON	0,005	0,01	√		√	
238	TRIFLURALIN	0,005	0,01		√		√
239	TRIFORINE	0,005	0,01	√		√	
240	UNICONAZOLE	0,005	0,01	√		√	
241	VAMIDOTHION	0,005	0,01	√		√	
242	VINCLOZOLIN	0,005	0,01		√		√
